# Scalable collective Lamb shift of a 1D superconducting qubit array in front of a mirror

**DOI:** 10.1038/s41598-019-55545-5

**Published:** 2019-12-16

**Authors:** Kuan-Ting Lin, Ting Hsu, Chen-Yu Lee, Io-Chun Hoi, Guin-Dar Lin

**Affiliations:** 1 Centre for Quantum Science and Engineering, Department of Physics, National Taiwan University, Taipei, 10617 Taiwan; 2 Centre for Quantum Technology and Department of Physics, National Tsing Hua University, Hsinchu, 30013 Taiwan

**Keywords:** Qubits, Quantum optics

## Abstract

We theoretically investigate resonant dipole-dipole interaction (RDDI) between artificial atoms in a 1D geometry, implemented by *N* transmon qubits coupled through a transmission line. Similar to the atomic cases, RDDI comes from exchange of virtual photons of the continuous modes, and causes the so-called collective Lamb shift (CLS). To probe the shift, we effectively set one end of the transmission line as a mirror, and examine the reflection spectrum of the probe field from the other end. Our calculation shows that when a qubit is placed at the node of the standing wave formed by the incident and reflected waves, even though it is considered to be decoupled from the field, it results in large energy splitting in the spectral profile of a resonant qubit located at an antinode. This directly implies the interplay of virtual photon processes and explicitly signals the CLS. We further derive a master equation to describe the system, which can take into account mismatch of participating qubits and dephasing effects. Our calculation also demonstrates the superradiant and subradiant nature of the atomic states, and how the CLS scales when more qubits are involved.

## Introduction

One of the intriguing phenomena of quantum electrodynamics is the emergence of the Lamb shift, which was first discovered by Lamb in 1947^1^, corresponding to the energy difference between 2S_1/2_ and 2P_1/2_ levels of a hydrogen atom. The understanding of such a shift opened up a new chapter of physics now known as quantum field theory, bringing in a concept that quantum vacuum must be treated as a zero-point state of numerous harmonic oscillators (photon modes), and quantum fluctuations allow both real and virtual processes to have physical effects. This perspective of quantum vacuum also plays an essential role in various scenarios such as spontaneous decay emission, squeezed vacuum states^2,3^, and the Casimir effect^4–6^. Recently, resonant dipole-dipole interaction (RDDI) mediated via exchange of virtual photons between multiple atoms has become one of the most interesting topics in, for instance, light scattering^7–9^ and coherent excitation transfer^10,11^ in atomic ensembles or structured arrays, atomic clocks^12^, topological quantum optics^13^, and quantum information processing^14^. Such RDDI results in the collective version of Lamb shift, sometimes also termed the cooperative Lamb shift (CLS) due to its close connection to cooperative phenomena such as super- and subradiance^15–17^. For past few years, CLS regarding atomic systems have been experimentally demonstrated and studied in atomic clouds^18–20^, nano-layer gases^21,22^, ensembles of nuclei^23^, and trapped ions^24^. Main challenges of observing CLS in atomic systems originate from vacuum mediated coupling weakened very fast as separation increases in 3D space. In order to probe the shift, ideally atoms must be placed at a distance comparable to the transition wavelength, or inside cavities or waveguides where field can be confined or directed, thus enhancing the interaction strength. Such consideration suggests that the circuit quantum electrodynamical (circuit QED, or cQED) systems are a perfect test bed for observing cooperative phenomena.

**Figure 1:**
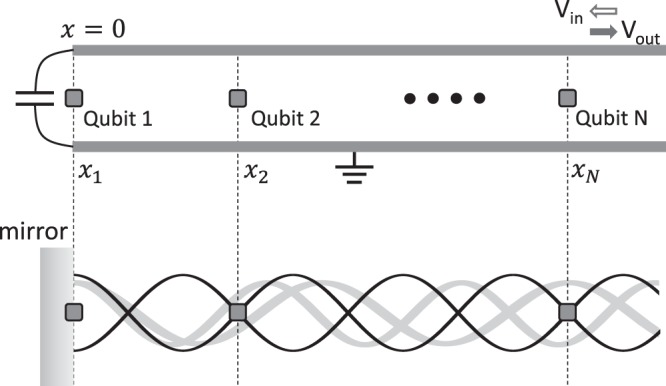
Architecture of the 1D array of transmon qubits coupled through a microwave waveguide, whose one end is terminated by a large capacitor at $x=0$, effectively serving as an antinode mirror. The probe field is fed from the other end of the waveguide, coherently superposes with the reflected field, forming a standing wave. When other qubits are placed at the nodes, they do not directly interact with probe photons. However, the qubits can still couple to other vacuum modes of continuous spectrum, mediating the RDDI only through virtual processes.

Circuit QED systems deal with artificial atoms coupled on-chip through waveguides. They are more easily fabricated to achieve the strong coupling or the superradiant regime compared to the atomic counterpart^25^, and have been used extensively to study the Tavis-Cummings model^26^, dipole-dipole coupling^27^, photon-ensemble interaction, super- and subradiance^28–32^, and quantum information oriented applications^33,34^. Up to present, the observation of CLS in cQED systems is still scarce except for a 2013 experiment^29^, where two superconducting qubits are both pumped in a 1D open waveguide, resulting in collective decay linewidth larger than the shift, seriously degrading the visibility of CLS. In order to resolve the tip shift from two very broad peaks, enormous times of data acquisition are required for a sufficient confidence level. Another way to look at the RDDI has been demonstrated in recent experiments with a few Rydberg atoms parted by a sub-wavelength distance with exchange interaction also in the microwave domain^11,35^. But instead of probing the CLS, they have measured Rabi-like excitation transfer between atoms, which demands both spatial and time-domain resolutions. In this work, we theoretically study the emergence of CLS by simply arranging a series of transmon qubits in front of a mirror, and probing for their reflection spectrum. Such arrangement has been realised with trapped atomic ions^36^ and superconducting qubits^37,38^, where the incident field is interfered with the reflected one, forming a standing wave. In the recent experiment^38^, we place one qubit at the antinode mirror while others at nodes with respect to their transition wavelength as shown in Fig. 1. This configuration is also closely connected to the nested structure of the giant atom proposal^39^. Interestingly, when a resonant field is fed from the open end, those node qubits seem to be decoupled from the probe and supposedly have no effect on the antinode qubit’s spectral profile through real photon exchange. This is however not the entire story because one neglects contributions from the whole range of vacuum modes that mediate RDDI without exchanging real photons. The advantage of insertion of a mirror is to introduce destructive interference that suppresses the collective decay linewidth, hence improving the visibility of the CLS. This distinguishes our scheme from open transmission line experiments whose measurement resolution is usually poor.

This work is devoted to thorough theoretical investigation from the fundamental theory to realistic experimental consideration^38^ such as dephasing and power broadening, as well as providing future guidance for scaling up the system and shift. In the following, we will presents an RDDI model based on a master-equation approach for our cQED system of a half-infinite waveguide. We will discuss the reflection spectral profiles and emergence of CLS associated with a two-qubit system, where the dephasing and power broadening effects will be studied to reflect the situations with real transmon artificial atoms. Finally, we will examine the scaling law of the CLS when more qubits are involved, for which we present an effective reduced scheme for both qualitative and quantitative explanations.

## Results

### Dipole-dipole interaction and the master equation

We consider a linear chain of *N* transmon qubits coupled to a common 1D waveguide whose one end is terminated by a very large capacitor. This amounts to setting the end as an antinode mirror regarding standing waves of this architecture. Different from a discrete spectrum in a cavity case with two mirrors, our system has a continuum of photon modes. The Hamiltonian describing this system can be written as $H={H}_{S}+{H}_{B}+{H}_{{int}}$^37,40–42^ with the atomic part ${H}_{S}={\sum }_{i}\,\hslash {\omega }_{i}{\sigma }_{i}^{+}{\sigma }_{i}^{-}$, the field part ${H}_{B}={\int }_{0}^{\infty }\,\hslash \omega {a}_{\omega }^{\dagger }{a}_{\omega }d\omega ,$ and the interaction under the rotating wave approximation ${H}_{{int}}=i\,{\sum }_{i}\,{\int }_{0}^{\infty }\,d\omega \hslash {g}_{i}(\omega )\,\cos ({k}_{\omega }{x}_{i}){a}_{\omega }{\sigma }_{i}^{+}+H.c.$ Here, ${\omega }_{i}$ denotes the transition frequency between the excited state $|e{\rangle }_{i}$ and the ground one $|g{\rangle }_{i}$ of the *i*th qubit located at *x*_*i*_, and ${\sigma }_{i}^{+}=|e{\rangle }_{i}\langle g|$ and ${\sigma }_{i}^{-}=|g{\rangle }_{i}\langle e|$ represent its raising and lowering operators, respectively, and *H*.*c*. denotes the Hermitian conjugate. The operator ${a}_{\omega }^{\dagger }$ (${a}_{\omega }$) creates (annihilates) a photon of frequency $\omega$, whose mode function is of the form ~$\cos \,{k}_{\omega }x$ due to the presence of the antinode mirror at $x=0$. The wavenumber ${k}_{\omega }=\omega /v$ with *v* the speed of light in the waveguide. Note that ${a}_{\omega }^{\dagger }$ and ${a}_{\omega }$ satisfy the commutation relation $[{a}_{\omega ^{\prime} },{a}_{\omega }^{\dagger }]=\delta (\omega -\omega ^{\prime} )$. Following the standard procedure to trace out the photonic degrees of freedom^43^ and applying the Born-Markov approximation, we arrive at the master equation

1 $$\begin{array}{rcl}\frac{d\rho }{dt} & = & i\,\sum _{i}\,{\delta }_{i}[{\sigma }_{i}^{+}{\sigma }_{i}^{-},\rho ]\\  &  & -\,i\,\sum _{ij}\,({\Delta }_{ij}^{+}-i{\gamma }_{ij}^{-})[{\sigma }_{i}^{+}{\sigma }_{j}^{-},\rho ]\\  &  & +\,i\,\sum _{i}\,{\Omega }_{p}^{i}\,\cos ({k}_{p}{x}_{i})[{\sigma }_{i}^{+}+{\sigma }_{i}^{-},\rho ]\\  &  & +\,\sum _{ij}\,({\gamma }_{ij}^{+}+i{\Delta }_{ij}^{-}){ {\mathcal L} }_{ij}[\rho ]\\  &  & +\,\sum _{i}\,{\gamma }_{i}^{\phi }{ {\mathcal L} }_{i}^{\phi }[\rho ].\end{array}$$

In this master equation, we have explicitly included a continuous-wave probe field incident from the other end of the waveguide with a detuning ${\delta }_{i}={\omega }_{p}-{\omega }_{i}$, with ${\omega }_{p}$ the probe light frequency, the associated Rabi frequency ${\Omega }_{p}^{i}$ seen by the *i*th qubit, and a wavenumber ${k}_{p}={\omega }_{p}/v$. The superoperator ${ {\mathcal L} }_{ij}[\rho ]\equiv 2{\sigma }_{j}^{-}\rho {\sigma }_{i}^{+}-{\sigma }_{i}^{+}{\sigma }_{j}^{-}\rho -\rho {\sigma }_{i}^{+}{\sigma }_{j}^{-}$ describes individual and cooperative dissipative processes. And ${ {\mathcal L} }_{i}^{\phi }[\rho ]\equiv 2{\sigma }_{i}^{ee}\rho {\sigma }_{i}^{ee}-{\sigma }_{i}^{ee}\rho -\rho {\sigma }_{i}^{ee}$ with ${\sigma }_{i}^{ee}=|e{\rangle }_{i}\langle e|$ is added by hand to account for individual pure dephasing characterised by ${\gamma }_{i}^{\phi }$. The dipole-dipole interaction, obtained by summing all contributions from the photon mode continuum, is now contained in ${\gamma }_{ij}^{\pm }=({\gamma }_{ij}\pm {\gamma }_{ji})/2$ and ${\Delta }_{ij}^{\pm }=({\Delta }_{ij}\pm {\Delta }_{ji})/2$ with

2 $${\gamma }_{ij}=\frac{{\gamma }_{ij}^{0}}{2}[\cos \,{k}_{j}({x}_{i}+{x}_{j})+\,\cos \,{k}_{j}|{x}_{i}-{x}_{j}|]$$

3 $${\Delta }_{ij}=\frac{{\gamma }_{ij}^{0}}{2}[\sin \,{k}_{j}({x}_{i}+{x}_{j})+\,\sin \,{k}_{j}|{x}_{i}-{x}_{j}|],$$

where ${\gamma }_{ij}^{0}\equiv \sqrt{{\gamma }_{i}({\omega }_{j}){\gamma }_{j}({\omega }_{j})}$ with the bare decay rate ${\gamma }_{i}=\pi {g}_{i}^{2}({\omega }_{j})$ evaluated at the *j*th qubit’s transition frequency ${\omega }_{j}$, and ${k}_{j}={\omega }_{j}/v$.

Here are a few remarks regarding the forms of Eqs. (2) and (3). First, for an open waveguide without a mirror, it can be proven that the dipole-dipole interaction between the *i*th and *j*th qubits depends only on the relative distance $|{x}_{i}-{x}_{j}|$^25^. The mirror effectively places image qubits on the other side of the mirror. Therefore qubit *i* does not only see the real qubit *j* at a distance $|{x}_{i}-{x}_{j}|$ but also the image one at distance $({x}_{i}+{x}_{j})$. Note that, in general, ${\gamma }_{ij}^{\pm }$ and ${\Delta }_{ij}^{\pm }$ can be finite with non-identical qubits, leading to non-Lindblad behaviour^44^. For identical qubits where the sub-indices are interchangeable, ${\gamma }_{ij}^{-}$ and ${\Delta }_{ij}^{-}$ vanish and hence the master equation retains the Lindblad form. We will see that Δ_*ij*_ then directly contributes to the CLS splitting.

### Reflection spectrum for two atoms

In order to probe the CLS configuration, we feed the probe signal from and acquire its reflection spectrum on the open end. Following the derivation summarised in the Methods section, we have the reflection amplitude

4 $$r=|1+i\,\mathop{\sum }\limits_{i=1}^{N}\,\frac{2{\eta }_{Ni}{\gamma }_{i}}{{\Omega }_{p}^{N}}\,\cos \,{k}_{p}{x}_{i}\langle {\sigma }_{i}^{-}\rangle |,$$

with ${\eta }_{Ni}={({E}_{J}^{(N)}{E}_{c}^{(i)}/{E}_{J}^{(i)}{E}_{c}^{(N)})}^{1/4}{\beta }_{N}/{\beta }_{i}$, where ${E}_{J}^{(i)}$ and ${E}_{C}^{(i)}$ are the Josephson energy and the charging energy, respectively, of the *i*th qubit; ${\beta }_{i}={C}_{C}^{i}/{C}_{T}^{i}$ is the ratio between the capacitor ${C}_{C}^{i}$ of the transmission line and the total capacitor ${C}_{T}^{i}$. The atomic variables $\langle {\sigma }_{i}^{-}\rangle$ needs to be solved by evaluating the master Eq. (1).

**Figure 2:**
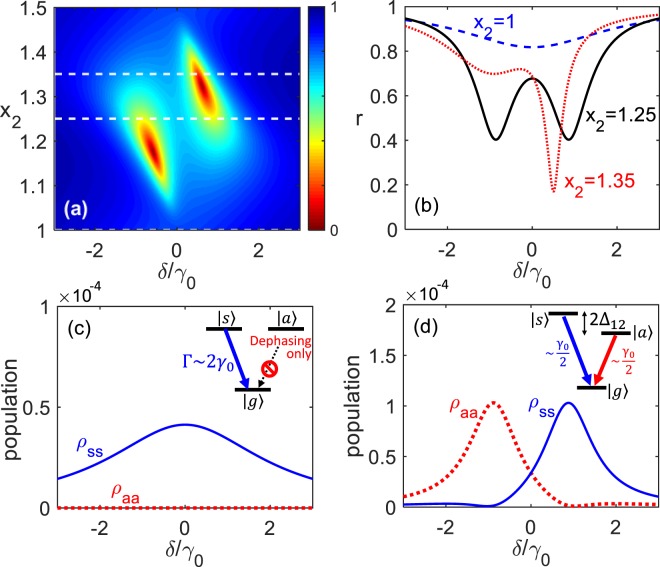
(**a**) Reflection spectrum for various ${x}_{2}$ in units of $\lambda$ with ${x}_{1}=0$. (**b**) The profiles corresponding to three white dashed line cuts in (**a**). For ${x}_{2}/\lambda =1$ (antinode), the spectral profile presents a single wide dip, signalling the superradiant nature. For ${x}_{2}/\lambda =1.25$ (node), the symmetric and antisymmetric states are split due to the CLS so that two dips merge corresponding to two resonant conditions. For ${x}_{2}$ away from the antinode, two dips move to the side of red detuning with the left one rising and finally fading out, and the right one moving toward the middle, and finally becoming superradiant as ${x}_{2}$ reaches the next antinode. (**c**) Population as a function of detuning in the symmetric (${\rho }_{ss}$) and antisymmetric (${\rho }_{aa}$) states for ${x}_{2}/\lambda =1$. (**d**) Similar to (**c**) but for ${x}_{2}/\lambda =1.25$. Note that for $1.5\le {x}_{2}/\lambda \le 2$, these curves are similar but with the roles of the symmetric and antisymmetric states are switched. (Other parameters: ${\gamma }^{\phi }=0.2{\gamma }_{0}$ and ${\Omega }_{p}=0.01{\gamma }_{0}$).

We start with discussion for the simplest case of two identical qubits, who share the same frequency and bare decay rate, ${\omega }_{1}={\omega }_{2}\equiv {\omega }_{0}$ and ${\gamma }_{12}^{0}={\gamma }_{21}^{0}\equiv {\gamma }_{0}$, respectively. In this case, ${\eta }_{21}=1$, ${\Delta }_{12}^{-}={\gamma }_{12}^{-}=0$, ${\Delta }_{12}^{+}={\varDelta }_{12}({x}_{1},{x}_{2})$ and ${\gamma }_{12}^{+}={\gamma }_{12}({x}_{1},{x}_{2})$ as functions of *x*_1_ and *x*_2_. Here, we set ${x}_{1}=0$, i.e., the 1st qubit is placed at the antinode mirror, and vary the position ${x}_{2}$ of the 2nd one. Since ${\gamma }_{12}$ and ${\Delta }_{12}$ are periodic functions of ${x}_{2}$, we will not lose generality if we only discuss the steady-state reflection spectrum from ${x}_{2}/\lambda =1$ (antinode) to ${x}_{2}/\lambda =1.5$ (next antinode) with $\lambda =2\pi v/{\omega }_{0}$, as shown in Fig. 2(a,b).

To understand the spectrum, it is instructional to perform analysis by recasting the master Eq. (1) into a non-Hermitian effective Hamiltonian:

5 $$\begin{array}{rcl}{H}_{eff}/\hslash  & = & -\sum _{i}\,({\delta }_{i}+i{\gamma }_{i}^{\phi }){\sigma }_{i}^{+}{\sigma }_{i}^{-}+\sum _{ij}\,({\Delta }_{ij}-i{\gamma }_{ij}){\sigma }_{i}^{+}{\sigma }_{j}^{-}\\  &  & -\,\sum _{i}\,{\Omega }_{p}^{i}\,\cos ({k}_{p}{x}_{i})({\sigma }_{i}^{+}+{\sigma }_{i}^{-}).\end{array}$$

For $N=2$, we consider a quantum state $|\psi \rangle ={c}_{ee}|ee\rangle +{c}_{eg}|eg\rangle +{c}_{ge}|ge\rangle +{c}_{gg}|gg\rangle$, whose dynamics follows the Schrodinger equation $i\hslash \frac{d}{dt}|\psi \rangle ={H}_{eff}|\psi \rangle$. Under the weak-field approximation, where we take ${c}_{gg}\sim 1$, ${c}_{ee}\sim 0$, the steady-state solution can be directly computed, and then from Eq. (4) the reflection amplitude. In the following, we will pay our special attention to the two exemplary cases with (i) ${x}_{2}/\lambda =1$ (antinode) and (ii) ${x}_{2}/\lambda =1.25$ (node) while keeping ${x}_{1}=0$.

In the case when ${x}_{2}=\lambda$, and setting the dephasing rate ${\gamma }_{1}^{\phi }={\gamma }_{2}^{\phi }={\gamma }^{\phi }$, the reflection spectrum is given by

6 $$r=|1-\frac{4{\gamma }_{0}({\gamma }^{\phi }-i\delta )}{2{\gamma }_{0}{\gamma }^{\phi }+{\gamma }^{\phi 2}-{\delta }^{2}-2i\delta ({\gamma }_{0}+{\gamma }^{\phi })}|.$$

When ${\gamma }^{\phi }$ is negligible, *r* approaches to ${|1-\frac{4{\gamma }_{0}{\gamma }^{\phi }}{{\delta }^{2}+4{\gamma }_{0}^{2}}|}^{1/2}$, forming a central dip of width $\Gamma =2{\gamma }_{0}$. This corresponds to the Dicke superradiant condition, where the linewidth is broaden by a factor of 2 for two qubits. By projecting the system to the symmetric state $|s\rangle =(|eg\rangle +|ge\rangle )/\sqrt{2}$ and the antisymmetric one $|a\rangle =(|eg\rangle -|ge\rangle )/\sqrt{2}$, one can see significant population in $|s\rangle$ with $|a\rangle$ almost depleted, as shown in Fig. 2(c). Note that when x_2_/λ = 1.5x_2_/λ = 1.5, the roles of the symmetric and antisymmetric states are switched because the distant qubit flips its phase due to the factor $\cos \,{k}_{p}{x}_{2}$, making ${\sigma }_{2}^{\pm }\leftrightarrow -\,{\sigma }_{2}^{\pm }$ and hence $|ge\rangle \leftrightarrow -\,|ge\rangle$.

For ${x}_{2}=1.25\lambda$, ${\Delta }_{12}={\gamma }_{0}$, similar analysis leads to

7 $$r=|1-\frac{2{\gamma }_{0}({\gamma }_{2}^{\phi }-i\delta )}{({\gamma }_{0}+{\gamma }_{1}^{\phi }){\gamma }_{2}^{\phi }-({\delta }^{2}-{\Delta }_{12}^{2})-2i\delta {\gamma }^{+}}|,$$

where γ+ = (γ0 + γϕ1 + γϕ2 )/2γ+ = (γ0 + γϕ1 + γϕ2 )/2. For small ${\gamma }_{2}^{\phi }/{\gamma }_{1}^{\phi }$, two dips correspond to $\delta \to {\delta }_{\pm }$ with

8 $${\delta }_{\pm }\approx \pm \,{\Delta }_{12}[1-\frac{{\gamma }_{0}^{2}-{\gamma }_{1}^{\phi 2}}{4{\Delta }_{12}^{2}}\frac{{\gamma }_{2}^{\phi }}{{\gamma }_{1}^{\phi }}]\to \pm \,{\Delta }_{12}$$

as ${\gamma }_{2}^{\phi }\to 0$. This suggests that Δ_12_ contributes to a coupling between $|s\rangle$ and $|a\rangle$ and splits the two states. Therefore, the exchange interaction results in the spectral splitting ${\delta }_{split}\equiv \mathrm{2|}{\delta }_{\pm }|\approx 2{\Delta }_{12}$ emerging in the the reflection profile. Such splitting has been clearly measured in the experiments^38^ with very good agreement to the theory. Finally, note that at $\delta =-\,{\Delta }_{12}$, ${\rho }_{ss}\approx \frac{|{\Omega }_{p}{|}^{2}{\gamma }_{2}^{\phi 2}}{2{\Delta }_{12}^{2}{\gamma }_{0}^{2}}\to 0$ as ${\gamma }_{2}^{\phi }\to 0$, implying that all the excitation is in state $|a\rangle$. Conversely, at $\delta =+\,{\Delta }_{12},$ only state $|s\rangle$ is populated. See Fig. 2(d). In the case of ${x}_{2}/\lambda =1.75$, the roles of the symmetric and antisymmetric states are switched due to the same argument in the case of ${x}_{2}/\lambda =1.5$ discussed previously.

A remarkable feature from examining Eq. (7) is that the linewidth of the dips is about ${\gamma }^{+}\approx \frac{1}{2}({\gamma }_{0}+{\gamma }_{1}^{\phi })$, which is smaller than ${\delta }_{split}\approx 2{\gamma }_{0}$, as long as ${\gamma }_{2}^{\phi }\ll {\gamma }_{0}$. This feature makes our mirror scheme distinguishable from the open transmission line experiment^29^ and other experiments with atomic ensembles^11,35^. The insertion of a mirror introduces image qubits that bring in phase relations leading to suppression of the collective linewidth without scaling up with the number of qubits.

### Dephasing and power broadening

We now examine the effect of dephasing on the splitting feature. Intuitively speaking, dephasing usually introduces broadening that degrades the quantum effects from being observed. In our case, it is however the individual dephasing, especially that of the mirror qubit, that makes the splitting visible. If we take ${\gamma }_{1}^{\phi }={\gamma }_{2}^{\phi }=0$, Eq. (7) gives $r=1$ constant reflection amplitude for any finite detuning *δ*. Therefore the splitting information is hidden. In fact, we need ${\gamma }_{1}^{\phi } > 0$ in order to view splitting as a trace of CLS from the reflection spectrum. We have shown in Eq. (8) that ${\delta }_{\pm }\to \pm \,{\Delta }_{12}$ as ${\gamma }_{2}^{\phi }\to 0$ for any ${\gamma }_{1}^{\phi } > 0$. When ${\gamma }_{2}^{\phi } > 0$, we find that the mismatch between ${\delta }_{split}$ and $2{\Delta }_{12}$ has a leading-order term proportional to ${\gamma }_{2}^{\phi }/{\gamma }_{1}^{\phi }$, which suggests that ${\delta }_{\pm }\to \pm \,{\Delta }_{12}$ as long as ${\gamma }_{2}^{\phi }/{\gamma }_{1}^{\phi }$ is small. The unit reflection amplitude in the case of no dephasing suggests that ${V}_{out}$ only differs from ${V}_{in}$ only by a pure phase factor as suggested by Eq. (10). But in the presence of dephasing, the phase relation between the input ${V}_{in}$ and the scattered component ${V}_{out}-{V}_{in}$ has been impaired, revealing the spectral landscape of the scattered signal.

**Figure 3:**
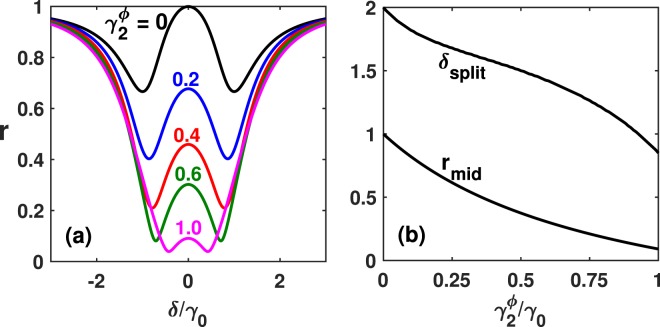
(**a**) Reflection spectrum for various dephasing rates of the 2nd qubit at ${x}_{2}/\lambda =1.25$. Here we set ${\gamma }_{1}^{\phi }=0.2{\gamma }_{0}$ and ${\Omega }_{p}=0.01{\gamma }_{0}$. (**b**) Spectral splitting ${\delta }_{split}$ in units of ${\gamma }_{0}$ and the height of the central maximum ${r}_{mid}$ as monotonically descending functions of the 2nd qubit’s dephasing rate ${\gamma }_{2}^{\phi }$.

Figure 3 shows our numerical calculation when ${\gamma }_{1}^{\phi }=0.2{\gamma }_{0}$ is fixed, corresponding to a typical experimental realisation. When ${\gamma }_{2}^{\phi }$ increases from zero, we find ${\delta }_{split}$ decreases monotonically from $2{\Delta }_{12}$. Another interesting feature regarding visibility of CLS is the central maximum ${r}_{mid}\equiv r(\delta =\mathrm{0)}$, which is also lowered with increasing ${\gamma }_{2}^{\phi }$ according to

9 $${r}_{mid}=1-\frac{2{\gamma }_{0}{\gamma }_{2}^{\phi }}{({\gamma }_{0}+{\gamma }_{1}^{\phi }){\gamma }_{2}^{\phi }+{\Delta }_{12}^{2}}.$$

In real experiments^38^, this maximum is always smaller than unity, reflecting the presence of dephasing mechanisms on the 2nd qubit. We find that ${r}_{mid}$ is dominantly determined by ${\gamma }_{2}^{\phi }$ and insensitive to ${\gamma }_{1}^{\phi }$ according to Eq. (9). Thus ${r}_{mid}$ provides a very good indication to be used to extract ${\gamma }_{2}^{\phi }$ without knowing the exact value of ${\gamma }_{1}^{\phi }$. The ratio of ${\gamma }_{2}^{\phi }$ thus obtained to the actual value is ${\Delta }_{12}^{2}/({\Delta }_{12}^{2}+{\gamma }_{1}^{\phi }{\gamma }_{2}^{\phi })$. Therefore, for ${\gamma }_{1}^{\phi }$, ${\gamma }_{2}^{\phi }\sim 0.5{\gamma }_{0}$, the estimated value of ${\gamma }_{2}^{\phi }$ is 20% less than the actual one; for ${\gamma }_{1}^{\phi }$, ${\gamma }_{2}^{\phi }\sim 0.2{\gamma }_{0}$ as in a typical experiment, it becomes only 4% less.

**Figure 4:**
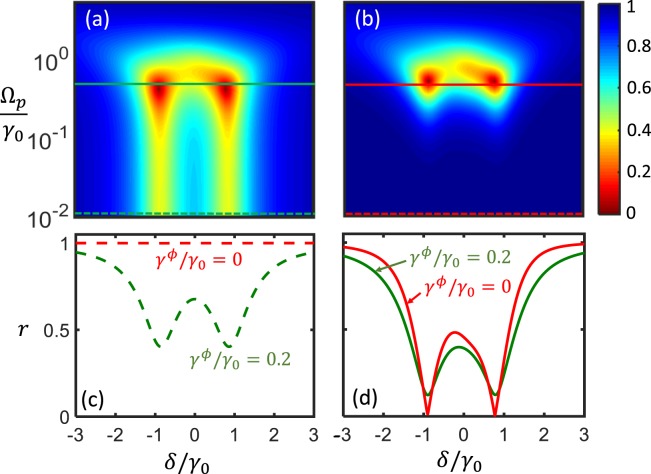
Power broadening of the reflection spectrum for Qubit 1 at ${x}_{1}=0$ and Qubit 2 at ${x}_{2}=1.25\lambda$ for (**a**) ${\gamma }^{\phi }=0.2{\gamma }_{0}$ and (**b**) ${\gamma }^{\phi }=0$. See text for other parameters. (**c** and **d**) Show the profiles for weak ${\Omega }_{p}=0.01{\gamma }_{0}$ and strong probing ${\Omega }_{p}=0.5{\gamma }_{0}$, respectively, corresponding to the dashed and solid linecuts, respectively, in (**a** and **b**).

Next, we discuss the cases when the probe power increases, where the effective-Hamiltonian approach breaks down at some point due to significant population in upper levels. By full density matrix calculation and inclusion of anharmonicity of the third level of the transmons, a power dependent reflection spectrum is shown in Fig. 4(a). Here we have plugged in typical parameters as in the experiment^38^ with ${\omega }_{1}={\omega }_{2}=2\pi \times 4.755\,{\rm{GHz}}$, ${x}_{1}=0$, ${x}_{2}=1.25\lambda$, and the wave speed $v=0.8948\times {10}^{8}$ m/s. The bare decay rate ${\gamma }_{0}=2\pi \times 17.2$ MHz, and then we have ${\gamma }_{11}={\Delta }_{12}={\gamma }_{0}$, ${\gamma }_{12}={\gamma }_{22}={\Delta }_{11}={\Delta }_{22}=0$, and ${\delta }_{split}\approx 2\pi \times 34$ MHz. The pure dephasing rates are taken the same for both the qubits ${\gamma }_{1}^{\phi }={\gamma }_{2}^{\phi }=0.2{\gamma }_{0}$. The anharmonicity defined as ${\omega }_{i}^{\ast }-{\omega }_{i}$ is −$20{\gamma }_{0}$, where ${\omega }_{i}^{\ast }$ is the frequency spacing between the next higher level to $|e{\rangle }_{i}$ of the *i*th atom. For weak probing ${\Omega }_{p}\lesssim 0.1{\gamma }_{0}$, the spectrum profiles remain independent of the probe power, reflecting the fact that the CLS originates from vacuum nature instead of the external field. As ${\Omega }_{p}$ increases, the green curves in Fig. 4(c,d) display clear power broadening of the two dips due to significant population in the second and third levels. In fact, the role of the third level is almost negligible as long as the anharmonicity is greater than $5{\gamma }_{0}$ given ${\Omega }_{p}\lesssim 0.5{\gamma }_{0}$. But with stronger probing field $0.5{\gamma }_{0} < {\Omega }_{p} < 2{\gamma }_{0}$, the spectral profile starts to show slight asymmetry because the third level is differently populated at different detuning. For ${\Omega }_{p}\gtrsim 2{\gamma }_{0}$, the system becomes saturated and attains unit reflection amplitude.

As a comparison, we also plot the cases with zero dephasing ${\gamma }^{\phi }=0$ in Fig. 4(b). We find that in this case the reflection amplitude under weak probing retains unity as shown by the red curve in Fig. 4(c). Interestingly, strong probing leads to power broadened linewidths for both qubits, recovering the profile of two-dip structure (represented by the red curve in Fig. 4(d)).

### Multi-atom cases

**Figure 5:**
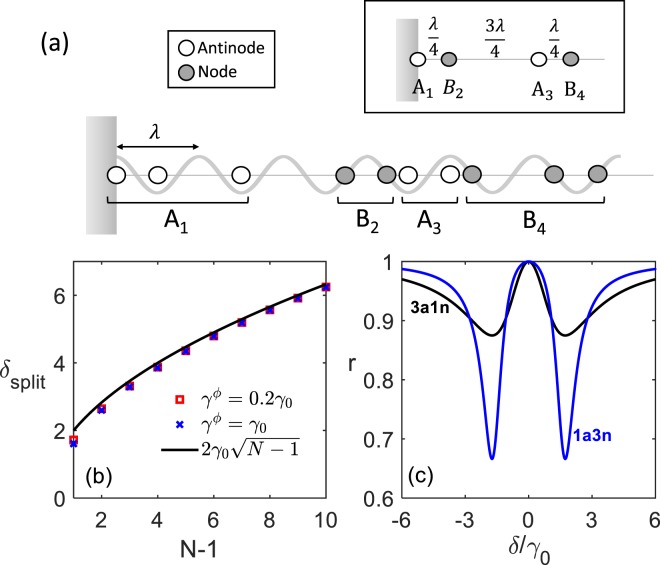
(**a**) Array of qubits located at either nodes and antinodes. The inset is the equivalent reduced scheme of “joint atoms” arranged at antinodes and nodes alternatively. (**b**) CLS splitting ${\delta }_{split}\approx 2{\gamma }_{0}\sqrt{N-1}$ for an qubit array of one at the mirror (${x}_{1}=0$) and $N-1$ ones at nodes. Small deviations can be observed with finite dephasing rate ${\gamma }^{\phi }$ for all the qubits. (**c**) Spectral profiles for three antinode qubits plus one node qubit (3a1n) and one antinode qubit plus three node ones (1a3n).

We now consider multi-atom cases with $N\ge 3$. We here focus on configurations with identical qubits either at antinodes or nodes as shown in Fig. 5(a). For analysis, we first take those qubits at antinodes/nodes in a row as a group. By doing so, the system now consists of antinode groups (${A}_{j}$) and node ones (${B}_{j}$) placed in alternative order, i.e., ${A}_{1}{B}_{2}{A}_{3}{B}_{4}\cdots$. For each antinode group ${A}_{j}$, we define the collective operator as ${S}_{j}^{\pm }\equiv \frac{1}{\sqrt{{n}_{j}}}\,{\sum }_{i\in {A}_{j}}\,{(-1)}^{2{x}_{i}/\lambda }{\sigma }_{i}^{\pm }$, and for each node one ${B}_{j}$, ${S}_{j}^{\pm }=\frac{1}{\sqrt{{n}_{j}}}\,{\sum }_{i\in {B}_{j}}\,{(-1)}^{2{x}_{i}/\lambda -1/2}{\sigma }_{i}^{\pm }$. Under the weak field approximation with only single excitation allowed, we show that these ${S}^{\pm }$’s become effective two-level spin operators by defining ${S}_{j}^{+}\equiv |a{\rangle }_{{A}_{j}}\langle g{|}^{\otimes {n}_{j}}$ for group ${A}_{j}$, and ${S}_{j}^{+}\equiv |b{\rangle }_{{B}_{j}}\langle g{|}^{\otimes {n}_{j}}$ for group ${B}_{j}$ (${S}_{{A}_{j},{B}_{j}}^{-}$ are their Hermitian conjugates), where $|a{\rangle }_{{A}_{j}}$ and $|b{\rangle }_{{B}_{j}}$ are collective excited states of ${A}_{j}$ and ${B}_{j}$, respectively, with ${n}_{j}$ the number of qubits in the group. For instance, for an antinode group ${A}_{1}=\{{x}_{1},{x}_{2},{x}_{3}\}=\{0,\lambda ,3\lambda /2\}$, ${S}_{1}^{+}=\frac{1}{\sqrt{3}}({\sigma }_{1}^{+}+{\sigma }_{2}^{+}-{\sigma }_{3}^{+})$ and $|a{\rangle }_{{A}_{1}}=\frac{1}{\sqrt{3}}(|e,g,g\rangle +|g,e,g\rangle -|g,g,e\rangle )$. Each group can then be seen as an effective two-level “joint atom” represented by the inset of Fig. 5(a).

To illustrate the “joint atom” picture, we go back to the effective Hamiltonian (5). We take identical qubits with the same dephasing rate ${\gamma }^{\phi }$ and probe detuning $\delta$ for simplicity. To avoid confusion hereafter, we denote the qubit index by $i$ or *i*′, and the joint atom index by $j$ or *j*′. Then the atom-probe interaction is given by ${\Omega }_{p}\,{\sum }_{i}\,\cos \,{k}_{p}{x}_{i}({\sigma }_{i}^{+}+{\sigma }_{i}^{-})\to {\Omega }_{p}\,{\sum }_{{A}_{j}}\,\sqrt{{n}_{j}}({S}_{j}^{+}+{S}_{j}^{-})$ with ${k}_{p}\approx {k}_{0}$, which can be seen as the joint atoms interacting with the probe field effectively. The dipole-dipole interaction characterised by the decay terms correspond to ${\sum }_{ii^{\prime} }\,{\gamma }_{ii^{\prime} }{\sigma }_{i}^{+}{\sigma }_{i^{\prime} }^{-}\to {\sum }_{{A}_{j}}\,{n}_{j}{\gamma }_{0}{S}_{j}^{+}{S}_{j}^{-}+{\sum }_{{A}_{j},{A}_{j^{\prime} }}\,\sqrt{{n}_{j}{n}_{j^{\prime} }}{\gamma }_{0}{S}_{j}^{+}{S}_{j^{\prime} }^{-}$ since there is no such contribution from pairs ($i\in {A}_{j}$, $i^{\prime} \in {B}_{j^{\prime} }$) and ($i\in {B}_{j}$, $i^{\prime} \in {B}_{j^{\prime} }$). The first terms correspond to superradiant decay of ${A}_{j}$, and the second terms correspond to the mutual decay between different joint atoms ${A}_{j}$ and ${A}_{{j}^{\text{'}}}$. Note that this analysis also suggests that $|a{\rangle }_{{A}_{j}}$ is superradiant with the enhanced decay rate ${n}_{j}{\gamma }_{0}$. Similarly, the dipole-dipole interaction characterised by exchange is given by ${\sum }_{ii^{\prime} }\,{\Delta }_{ii^{\prime} }{\sigma }_{i}^{+}{\sigma }_{i^{\prime} }^{-}\to {\sum }_{{A}_{j},{B}_{j^{\prime} }}\,\sqrt{{n}_{j}{n}_{j^{\prime} }}{\gamma }_{0}{S}_{j}^{+}{S}_{j^{\prime} }^{-}$ for $j < j^{\prime}$. Note that the contributions are all zero from pairs ($i\in {A}_{j}$, $i^{\prime} \in {A}_{j^{\prime} }$), ($i\in {B}_{j}$, $i^{\prime} \in {B}_{j^{\prime} }$), and ($i\in {B}_{j}$, $i^{\prime} \in {A}_{j^{\prime} }$) for $j^{\prime}  > j$. This is equivalent to re-scale the coupling strength by a factor $\sqrt{{n}_{j}{n}_{j^{\prime} }}$, the square root of the product of the qubit numbers of two joint atoms ${A}_{j}$ and ${B}_{j^{\prime} }$. We can then take an effective reduced scheme with ${A}_{j}$ located at $(j-\mathrm{1)}\lambda$ and ${B}_{j^{\prime} }$ at $(j^{\prime} -3/4)\lambda$ as represented by Fig. 5(a), which will yield almost the same spectral landscape as the inset in Fig. 5(a).

Note that, for the joint atom ${B}_{j^{\prime} }$, the effective spontaneous emission rate ($|b{\rangle }_{{B}_{j^{\prime} }}\to |g{\rangle }^{\otimes {n}_{j^{\prime} }}$) remains zero since every qubit in this group sits at the node. This implies that no spontaneous linewidth of ${B}_{j^{\prime} }$ contributes to the linewidth of the CLS splitting signal. Consider the case of an array consisting of two groups ${A}_{1}$ and ${B}_{2}$ only, with ${n}_{1}$ antinode and ${n}_{2}$ node qubits, respectively. It can be viewed as a joint atom ${A}_{1}$ placed at the mirror is of spontaneous linewidth ${n}_{1}{\gamma }_{0}$, and another joint atom ${B}_{2}$ at $\mathrm{1/4}\lambda$ with no such linewidth. There exists an exchange coupling ${\Delta }_{j=1,j^{\prime} =2}=\sqrt{{n}_{1}{n}_{2}}{\gamma }_{0}$ between them. The CLS splitting is thus given by ${\delta }_{split}\approx 2\sqrt{{n}_{1}{n}_{2}}{\gamma }_{0}$. Figure 5(b) presents the scaling law of ${\delta }_{split}$, which indeed agrees with the above analysis. Small deviation is visible but negligible when dephasing is included, and diminishes as $N$ becomes large. In Fig. 5(c), we compare the reflection spectral profiles of two situations: $\{{x}_{1},{x}_{2},{x}_{3},{x}_{4}\}=\{0,\lambda ,2\lambda ,9\lambda /4\}$ and $\{0,\lambda /4,3\lambda /4,5\lambda /4\}$. In the former case, $({n}_{1},{n}_{2})=(3,1)$ and the latter $({n}_{1},{n}_{2})=(1,3)$, the CLS splittings are the same $2\sqrt{3}{\gamma }_{0}$. The former has a broadened linewidth $3{\gamma }_{0}$ due to superradiant enhancement in ${A}_{1}$ while the linewidth of each dip in the latter case is still comparable to ${\gamma }_{0}$. The latter case shows exactly the beauty of the scheme with a mirror: Adding more node qubits $({n}_{2})$ in a row enhances the splitting without significantly broadening the signal dips (due to ${n}_{1}=1$), making the CLS signal to be spotted easily by simple reflection measurement.

## Conclusion

In summary, we have studied the dipole-dipole interaction between artificial atoms mediated by 1D vacuum modes in a waveguide. Setting one end of the waveguide to be a mirror, we can probe the collective Lamb shift by studying the reflection spectrum. When a qubit is placed at the node, we isolate it from coupling to other qubits through the resonant field. Instead, the exchange interaction remains effective via virtual photons, causing the collective Lamb splitting between symmetric and antisymmetric levels that can now be clearly visible by means of a very simple reflection measurement.

Our calculation highly agrees with the recent experimental results^38^. We have derived the master equation to describe general cases and given analytical expressions for certain circumstances. We have also investigated the effects of dephasing, power of probing, and the scaling law when more qubits are added. For special cases with many qubits placed only at antinodes and nodes, we have developed a reduced scheme under the weak field approximation, and explained the scaling behaviour.

For future outlook, we find close connection of our findings to recent work^39,45^, where atoms are considered large compared to the transition wavelength, and thought to have multiple chances of interaction before the field leaves. We expect similar analysis for some interesting interference effects, and our results can be very useful for quantum optical study and quantum simulation.

## Methods

As measured in many experiments^37,41,46,47^, the reflection amplitude is defined as

10 $$r(x,t)\equiv |\langle {V}_{out}(x,t)/{V}_{in}(x,t)\rangle |,$$

where the output signal ${V}_{out}(x,t)={V}_{in}(x,t)+{V}_{sc}(x,t)$ with the input voltage ${V}_{in}$ and scattered one ${V}_{sc}$. The input signal is assumed to be of the form

11 $${V}_{in}(x,t)={V}_{0}{e}^{i{k}_{p}r}$$

viewed from the rotating frame of the probe frequency, where ${V}_{0}$ is the amplitude of the input voltage with its corresponding wave number ${k}_{p}$. The scattered voltage can be calculated from the flux^41,48^

12 $$\Phi (x,t)=\sqrt{\frac{\hslash {Z}_{0}}{\pi }}\,\int \,\frac{d\omega }{\sqrt{\omega }}\,\cos \,{k}_{\omega }x({a}_{\omega }+{a}_{\omega }^{\dagger })\equiv {\Phi }^{out}+{\Phi }^{in}$$

with the characteristic impedance ${Z}_{0}$. Then the scattered signal is obtained by differentiating the outgoing-wave part ${V}_{sc}=\partial {\Phi }^{out}/\partial t$. In the probe-frequency frame,

13 $${V}_{sc}(x,t)=-\,i\sqrt{\frac{\hslash {Z}_{0}}{4\pi }}\,{\int }_{0}^{\infty }\,\sqrt{\omega }{\tilde{a}}_{\omega }(t){e}^{i{k}_{\omega }x-i(\omega -{\omega }_{p})t}d\omega .$$

Here we have used the fact that the field operator can be expressed in terms of the slowly-varying amplitude ${a}_{\omega }(t)={\tilde{a}}_{\omega }(t){e}^{-i\omega t}$ and ${\dot{\tilde{a}}}_{\omega }\approx 0$. Through the standard procedures, the photonic operator is related to the atomic one^49,50^

14 $${\tilde{a}}_{\omega }(t)=-\,\mathop{\sum }\limits_{i=1}^{N}\,{g}_{i}(\omega )\,{\int }_{0}^{t}\,{\tilde{\sigma }}_{i}^{-}(t^{\prime} ){e}^{i(\omega -{\omega }_{i})t^{\prime} }dt^{\prime} +{\rm{noise}},$$

where the atomic operator is also assumed of the form ${\sigma }_{i}^{-}(t)={\tilde{\sigma }}_{i}^{-}(t){e}^{-i{\omega }_{i}t}$. Note that the noise term will be omitted hereafter since it is averaged out in the vacuum state. Substituting Eq. (14) into Eq. (13), and using Eqs. (10) and (11), we then have the scattered signal and the reflection amplitude, respectively,

15 $${V}_{sc}=i\,\mathop{\sum }\limits_{i=1}^{N}\,\sqrt{\hslash \pi {Z}_{0}{\omega }_{i}}{g}_{i}({\omega }_{i}){\tilde{\sigma }}_{i}^{-},$$

16 $$r=|1+i\,\mathop{\sum }\limits_{i=1}^{N}\,\sqrt{\hslash \pi {Z}_{0}{\omega }_{i}}{g}_{i}({\omega }_{i})\,\cos \,{k}_{p}{x}_{i}\langle {\sigma }_{i}^{-}\rangle /{V}_{0}|.$$

The photon-atom coupling strength for transmon qubits is given by

17 $${g}_{i}(\omega )=e{\beta }_{i}{(\frac{{E}_{J}^{(i)}}{8{E}_{C}^{(i)}})}^{1/4}\sqrt{\frac{2{Z}_{0}\omega }{\pi \hslash }},$$

where *e* is the electron charge; ${Z}_{0}$ is the characteristic impedance of the transmission line; ${\beta }_{i}={C}_{C}^{i}/{C}_{T}^{i}$ is the ratio between the capacitor ${C}_{C}^{i}$ of the transmission line and the total capacitor ${C}_{T}^{i}$; ${E}_{J}^{(i)}$ and ${E}_{C}^{(i)}$ are the Josephson energy and the charging energy, respectively, of the $i$th qubit^40,51,52^. Note that the input voltage ${V}_{0}$ is viewed right outside the outmost qubit (the $N$th one), and is connected to the Rabi frequency via

18 $${V}_{0}=\frac{{\Omega }_{p}^{N}}{2{g}_{N}({\omega }_{N})}\sqrt{\frac{\hslash {Z}_{0}{\omega }_{N}}{\pi }}.$$

By expressing ${V}_{0}$ in terms of ${\Omega }_{p}$, we finally obtain the reflection amplitude

19 $$r=|1+i\,\mathop{\sum }\limits_{i=1}^{N}\,\frac{2{\eta }_{Ni}{\gamma }_{i}}{{\Omega }_{p}^{N}}\,\cos \,{k}_{p}{x}_{i}\langle {\sigma }_{i}^{-}\rangle |,$$

with $${\eta }_{Ni}={({E}_{J}^{(N)}{E}_{c}^{(i)}/{E}_{J}^{(i)}{E}_{c}^{(N)})}^{1/4}{\beta }_{N}/{\beta }_{i}$$.
